# Electron beam-induced deposition of platinum from Pt(CO)_2_Cl_2_ and Pt(CO)_2_Br_2_

**DOI:** 10.3762/bjnano.11.161

**Published:** 2020-11-27

**Authors:** Aya Mahgoub, Hang Lu, Rachel M Thorman, Konstantin Preradovic, Titel Jurca, Lisa McElwee-White, Howard Fairbrother, Cornelis W Hagen

**Affiliations:** 1 Delft University of Technology, Fac. Applied Sciences, Dept. Imaging Physics, Lorentzweg 1, 2628CJ Delft, Netherlands; 2 Department of Chemistry, University of Florida, Gainesville, Florida, 32611-7200, USA; 3 Department of Chemistry, Johns Hopkins University, Baltimore, MD, 21218, USA; 4 Department of Chemistry and the Renewable Energy and Chemical Transformations Cluster, University of Central Florida, Orlando, Florida, 32816-2366, USA

**Keywords:** energy-dispersive X-ray spectroscopy (EDX), focused electron beam-induced deposition (FEBID), nanofabrication, platinum precursors, scanning electron microscopy (SEM), thermogravimetric analysis (TGA)

## Abstract

Two platinum precursors, Pt(CO)_2_Cl_2_ and Pt(CO)_2_Br_2_, were designed for focused electron beam-induced deposition (FEBID) with the aim of producing platinum deposits of higher purity than those deposited from commercially available precursors. In this work, we present the first deposition experiments in a scanning electron microscope (SEM), wherein series of pillars were successfully grown from both precursors. The growth of the pillars was studied as a function of the electron dose and compared to deposits grown from the commercially available precursor MeCpPtMe_3_. The composition of the deposits was determined using energy-dispersive X-ray spectroscopy (EDX) and compared to the composition of deposits from MeCpPtMe_3_, as well as deposits made in an ultrahigh-vacuum (UHV) environment. A slight increase in metal content and a higher growth rate are achieved in the SEM for deposits from Pt(CO)_2_Cl_2_ compared to MeCpPtMe_3_. However, deposits made from Pt(CO)_2_Br_2_ show slightly less metal content and a lower growth rate compared to MeCpPtMe_3_. With both Pt(CO)_2_Cl_2_ and Pt(CO)_2_Br_2_, a marked difference in composition was found between deposits made in the SEM and deposits made in UHV. In addition to Pt, the UHV deposits contained halogen species and little or no carbon, while the SEM deposits contained only small amounts of halogen species but high carbon content. Results from this study highlight the effect that deposition conditions can have on the composition of deposits created by FEBID.

## Introduction

Focused electron beam-induced deposition (FEBID) is a direct-write nanopatterning technique. FEBID has very high design flexibility and does not require masks or resist and development. Moreover, it does not need to be performed in a clean room with multiple process stages, such as spin coating, deposition, development, and etching; it is a single step process [1]. The process starts by injecting a precursor gas into the vacuum chamber of an electron microscope [2–3]. At specific locations on the substrate exposed to the electron beam, the transiently adsorbed precursor molecules decompose, forming a deposit while the volatile byproducts of the reaction desorb into the vacuum [4–7].

One of the main challenges associated with FEBID is the typical low purity of the deposits. Many FEBID precursors are organometallic, leading to high carbon content in the deposit [6,8–9]. Often, unwanted fragments of the precursor molecules remain in the deposits [10]. Some precursors perform better in this respect but are thermally unstable, for instance, ClAuCO and ClAuPF_3_ [11–13]. Therefore, it is desirable to design new stable precursors that enable the deposition of pure metals. In this work, two novel platinum precursors (Pt(CO)_2_Cl_2_ and Pt(CO)_2_Br_2_) were synthesized and tested. Both Pt(CO)_2_X_2_ complexes were compared to the widely used commercially available precursor MeCpPtMe_3_. The design of Pt(CO)_2_X_2_ takes advantage of the known tendency for CO and halogens to dissociate from metal centres upon electron irradiation [14–16]. Other organometallic compounds that include a halide and CO ligands, such as (η^3^-C_3_H_5_)Ru(CO)_3_Br, showed the loss of CO upon electron irradiation [17–18], as have CO-containing precursors without halides such as W(CO)_6_ [19] and Co(CO)_3_NO [20]. In addition, the use of four-coordinate Pt(II) centres minimizes the number of metal–ligand bonds that need to be broken for complete precursor decomposition [14]. Electron-induced decomposition of adsorbed Pt(CO)_2_Cl_2_ has been previously studied using X-ray photoelectron spectroscopy (XPS) and mass spectrometry, and some deposits were produced in the ultrahigh vacuum (UHV) environment of an Auger electron spectroscopy (AES) setup [21–22]. In the work presented here, Pt(CO)_2_Cl_2_ and Pt(CO)_2_Br_2_ were used for FEBID in a regular scanning electron microscope (SEM) and Pt(CO)_2_Br_2_ was also used for deposition in the aforementioned AES setup. A comparison of the two precursors is interesting because the bromide derivative is more volatile than the chloride (vide infra), facilitating delivery to the substrate, although a study of the electron-induced reactivity of (η^3^-C_3_H_5_)Ru(CO)_3_Br and (η^3^-C_3_H_5_)Ru(CO)_3_Cl under UHV conditions demonstrated that the response of the halides to electron flux was similar for both compounds [17]. The typical issues to be addressed when testing novel precursors include: (i) precursor storage, (ii) gas injection system (GIS) loading, (iii) optimal precursor temperature for deposition, (iv) precursor volatility and transport from the SEM chamber, (v) ability of precursor to form solid deposits upon electron exposure, and (vi) deposition rate and deposit composition. We now report an investigation of these practical aspects of Pt(CO)_2_Cl_2_ and Pt(CO)_2_Br_2_ in the context of their potential use in FEBID of Pt nanostructures.

## Experimental

### Synthesis

**Pt(CO)_2_Cl_2_**. The compound was synthesized via a modified literature procedure [15,23]. A suspension of PtI_2_ (0.4 g, 0.9 mmol) in toluene (15 mL) was prepared in a Schlenk flask under N_2_ and CO was bubbled through the suspension for 2 h. SO_2_Cl_2_ (0.6 g, 4.5 mmol) was then added into the system. The reaction mixture was stirred at room temperature under N_2_ for six additional hours, during which the black suspension became a dark purple solution. Anhydrous *n*-heptane (30 mL) was added into the solution and the flask was stored in the freezer overnight. The product was obtained as pale white crystals. The solvent was removed by cannulation and the solid was washed with *n*-heptane until the washes were colourless. After drying under vacuum for several hours, the product was obtained as needle-shaped crystals (0.15 g, yield 52%). The compound was identified by comparison to literature data [24]. ^13^C NMR (C_6_D_6_): δ 151.01. IR: ν_CO_ 2127, 2171 cm^−1^. Pt(CO)_2_Cl_2_ sublimes at 35–40 °C at 125 ± 1 mTorr.

**Pt(CO)_2_Br_2_**. The compound was synthesized using a modified literature method [25]. PtBr_2_ (0.51 g) was stirred in 1,2-dichloroethane (42 mL) in a 300 mL Parr reactor with a glass liner for 1.5 h at room temperature under CO (150 psi). The Parr reactor was then heated to 70 °C using a water bath and the stirring was continued for another 3 h. After the reactor was cooled to room temperature, the reaction mixture was stirred overnight. After changing the atmosphere back to N_2_, the Parr reactor was opened in a glove box. The yellow-brown suspension was transferred into a Schlenk flask and the solvent was removed on a Schlenk line. A light yellow solid (0.47 g, yield 80%) was collected after purification by sublimation at 30–35 °C at 125 ± 1 mTorr. The compound was identified by comparison to literature data [25]. ^13^C NMR (CDCl_3_): δ 152.34. IR (CH_2_Cl_2_): ν_CO_ 2129, 2170 cm^−1^.

Both precursors are very sensitive to air and humidity and decompose immediately, discolouring to brown if exposed to air. For comparison, commercial samples of the commonly used Pt precursor MeCpPtMe_3_ are crystalline and colourless at room temperature and the compound is not sensitive to air or humidity.

### Thermogravimetric analysis

Thermogravimetric analysis (TGA) of Pt(CO)_2_X_2_ was conducted on an ISI TGA-1000 instrument housed inside a nitrogen-atmosphere glove box, using Pt sample pans, with a 5 cc/min flow of ultrahigh-purity N_2_. The complexes were measured non-isothermally at a steady ramp rate of 10 °C/min (sample masses: Pt(CO)_2_Cl_2_ 2.99 mg, Pt(CO)_2_Br_2_ 3.01 mg), and isothermally at 90 °C (sample masses: Pt(CO)_2_Cl_2_ 2.99 mg, Pt(CO)_2_Br_2_ 2.98 mg) with the following protocol: 25–90 °C at a ramp rate of 10 °C/min, hold 240 min @90 °C, 90–400 °C at a ramp rate of 10 °C/min.

### FEBID in the SEM

Deposition was performed in a Thermo Fisher Scientific (TFS) Nova Nano Lab 650 dual-beam system. Standard TFS gas injection systems (GIS) were used to introduce the new precursors into the SEM chamber, using a separate GIS for each precursor. Since Pt(CO)_2_Cl_2_ and Pt(CO)_2_Br_2_ are both very sensitive to O_2_ and H_2_O, they were stored in a nitrogen-filled glove box and GIS filling was carried out in the box. The GIS needles were positioned about 150 µm from the electron beam and about 150 µm above the sample surface, which was at the eucentric height (5 mm working distance) in all deposition experiments. This allows for some thermal expansion of the needle when the GIS is heated.

After installing each precursor-filled GIS, its crucible temperature was determined. The desired temperature should generate a pressure rise that is sufficient for deposition without exceeding the maximum pressure allowed in the SEM chamber (approximately 10^−4^ mbar).

A silicon substrate was used for all deposition experiments, patterned such that circular areas of pristine silicon are surrounded by black silicon (obtained by reactive ion etching). The black silicon area aids in focusing the electron beam close to the circular Si areas in which the deposition was done. Unless stated otherwise, the beam energy used during deposition was 18 kV and the beam current was varied from 12–140 pA between experiments. To achieve high spatial resolution, all deposition experiments were done in ultrahigh-resolution (immersion) mode. Specific patterning parameters such as electron beam dwell time and the refresh time between exposure passes will be detailed for each experiment. To characterize FEBID growth, the height and base diameter of pillars were measured using 35° tilt images.

### Energy dispersive X-ray analysis

Energy dispersive X-ray (EDX) spectroscopy was performed using an Oxford XMax150 detector on a Zeiss Supra 55 SEM. For EDX measurements, 250 × 250 nm^2^ squares were deposited, thick enough to minimize the signal from the Si substrate during the analysis with a 5 keV beam. The beam current during EDX was 5 nA and the sample was mounted at a working distance of 7.5 mm and tilted by 35° to maximise the EDX signal. The system was plasma cleaned before the EDX measurements were taken to minimize carbon contamination.

### UHV deposition

The UHV deposition experiments were performed by introducing the Pt(CO)_2_X_2_ precursor into a chamber (base pressure of 3 × 10^−9^ mbar) furnished with a PHI 610 Scanning Auger Microprobe system (LaB_6_ filament). The precursor was heated to 85–90 °C (Pt(CO)_2_Br_2_) or approx. 80 °C (Pt(CO)_2_Cl_2_) and leaked through an UHV-compatible leak valve equipped with a directional doser, which was used to increase the partial pressure of the precursor at the surface of the substrate (silicon in the case of Pt(CO)_2_Br_2_, Ru-capped Si/Mo multilayers in the case of Pt(CO)_2_Cl_2_). Deposits were produced over 12 h under steady-state deposition conditions using a 3 kV electron beam with a substrate current of 0.5–1.0 µA. A pressure of 5–7 × 10^−8^ mbar was maintained throughout deposition, with the substrate at room temperature. Deposits made under UHV conditions were imaged using a JEOL JSM-IT100 SEM with a 5 kV primary electron beam (8 nm resolution) and analysed using the JEOL-made EDX unit. While deposit thickness was not measured, UHV-deposited samples were thick enough to yield a minimal silicon substrate signal during EDX measurements.

## Results and Discussion

### Thermal properties of the precursor

**Figure 1:**
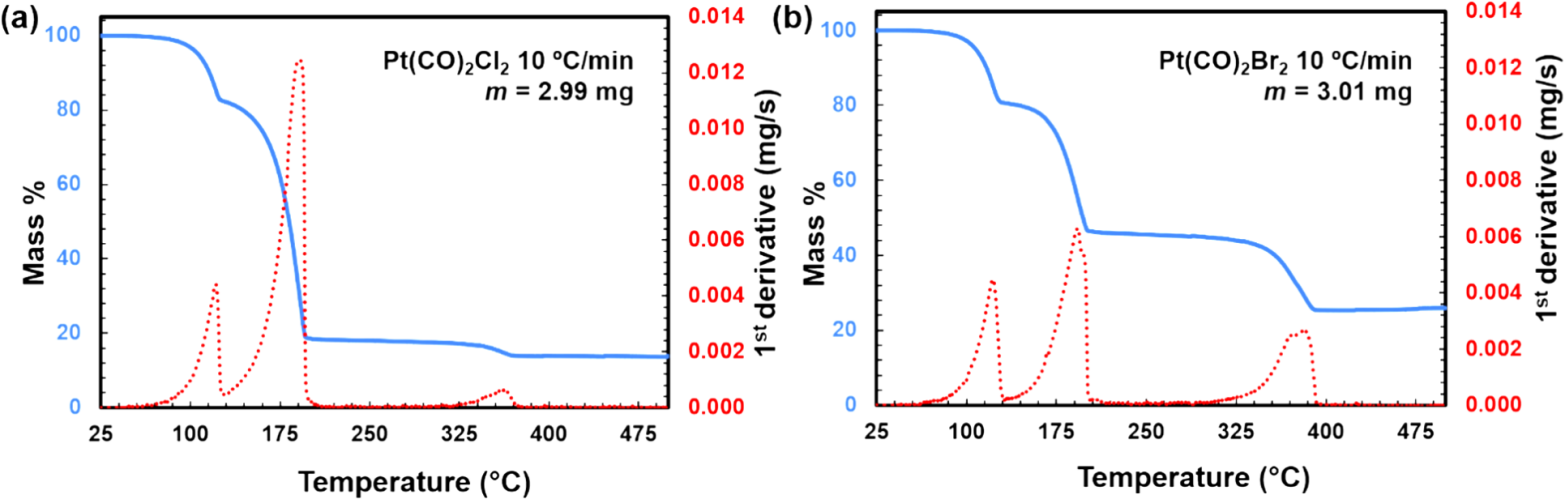
Thermogravimetric analysis (TGA) and 1st derivative DTG of (a) Pt(CO)_2_Cl_2_ and (b) Pt(CO)_2_Br_2_.

Thermogravimetric analysis was used to make a preliminary assessment of the thermal stability of Pt(CO)_2_Cl_2_ and to study its volatility at near-atmospheric pressure (approx. 1.2 atm positive pressure under N_2_). In the standard TGA experiment (Figure 1a), the compound underwent an initial mass loss beginning at roughly 80 °C, resulting in an intermediate residual mass of approximately 83% at 125 °C. This initial mass drop is consistent with the loss of both CO ligands to form PtCl_2_ (82.6% calculated residual mass). Additional mass loss occurred until approximately 200 °C was reached, leaving a residual mass of around 20%. This mass plateaued until 350 °C, at which point further mass loss ensued to yield a final residual mass of 14%, far below the initial Pt content of 60.5%. Pt(CO)_2_Br_2_ displayed a similar three-step mass loss (Figure 1b). Initial mass loss began at roughly 80 °C, resulting in an intermediate residual mass of approximately 80% at 125 °C. The second step did not occur until approximately 200 °C was reached, leaving a residual mass of around 46%. This mass plateaued until 330 °C at which point further mass loss ensued until a final residual mass of 26% was reached, significantly below the initial Pt content of 47.5%. The low final residual masses are characteristic of a combination of decomposition and sublimation during the experiment.

**Figure 2:**
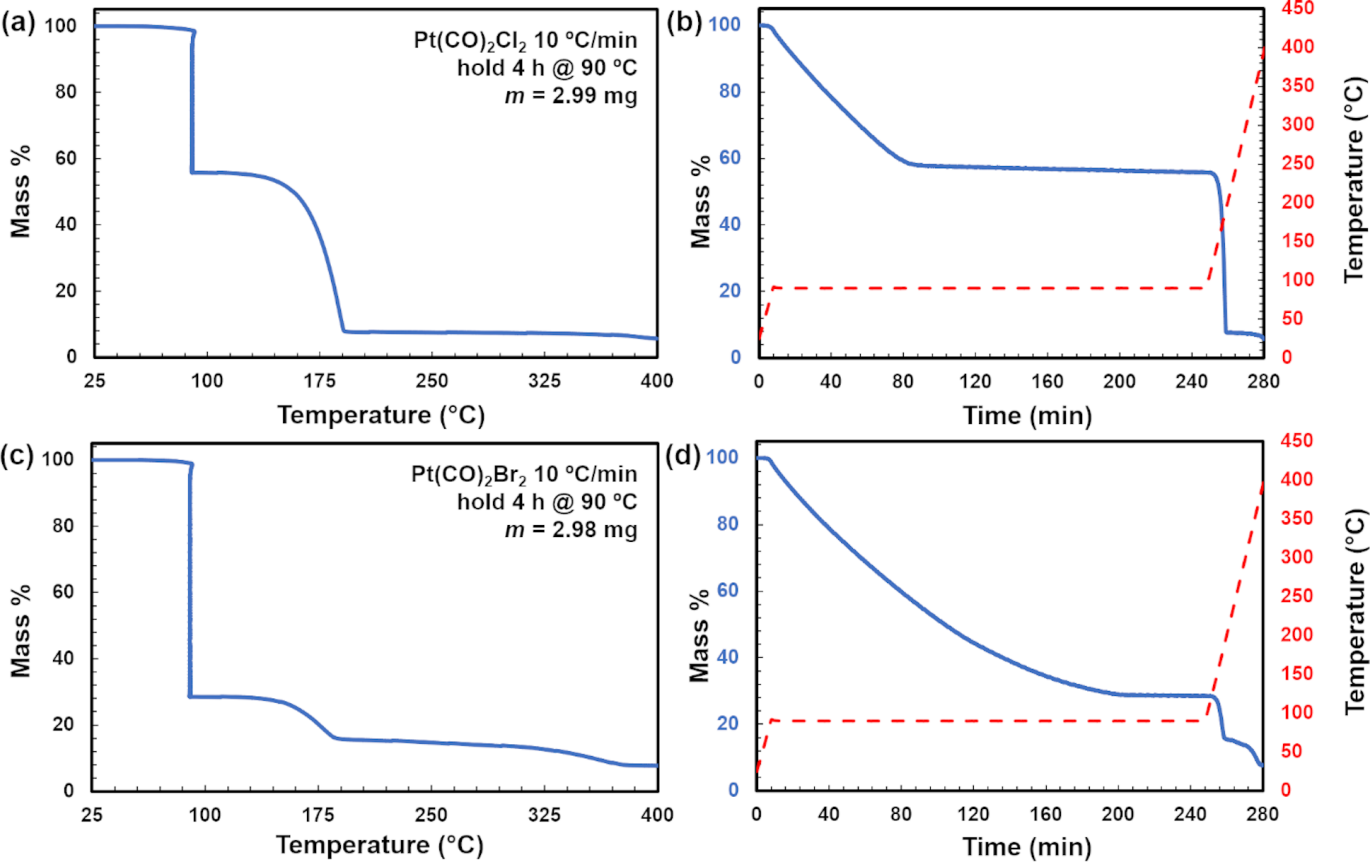
Isothermal TGA traces plotted as a function of temperature and time for (a,b) Pt(CO)_2_Cl_2_ and (c,d) Pt(CO)_2_Br_2_ for isothermal analysis at 90 °C for 4 h.

When data were obtained at an isothermal temperature of 90 °C (Figure 2), the mass losses were consistent with a mixture of sublimation and decomposition. For Pt(CO)_2_Cl_2_, a plateau corresponding to ca. 58% of original mass is likely indicative that prolonged heating at 90 °C will result in precursor decomposition (Figure 2a,b). Conversely, for Pt(CO)_2_Br_2_, prolonged heating at 90 °C facilitated a greater degree of sublimation vs decomposition whereupon approximately 29% of the original mass remained (Figure 2b,c). This is reasonably below the initial Pt content of 47.5%. The TGA studies indicate that Pt(CO)_2_Br_2_ is more stable under prolonged heating, leading to increased volatility, but Pt(CO)_2_Cl_2_ is more efficient under a faster ramped-temperature process (10 °C/min).

### Precursor gas properties

Precursor crucible temperature was determined for both precursors by gradually increasing the temperature of the GIS while monitoring the pressure in the SEM vacuum chamber until a pressure rise is observed, suitable for deposition and not exceeding the maximum allowable pressure in the SEM. Temperatures suitable for deposition were found to be 80 °C and 60 °C for Pt(CO)_2_Cl_2_ and Pt(CO)_2_Br_2_, respectively, with corresponding pressures in the SEM chamber of about 4 × 10^−6^ mbar. All SEM deposition experiments done with these precursors were carried out at these crucible temperatures.

**Figure 3:**
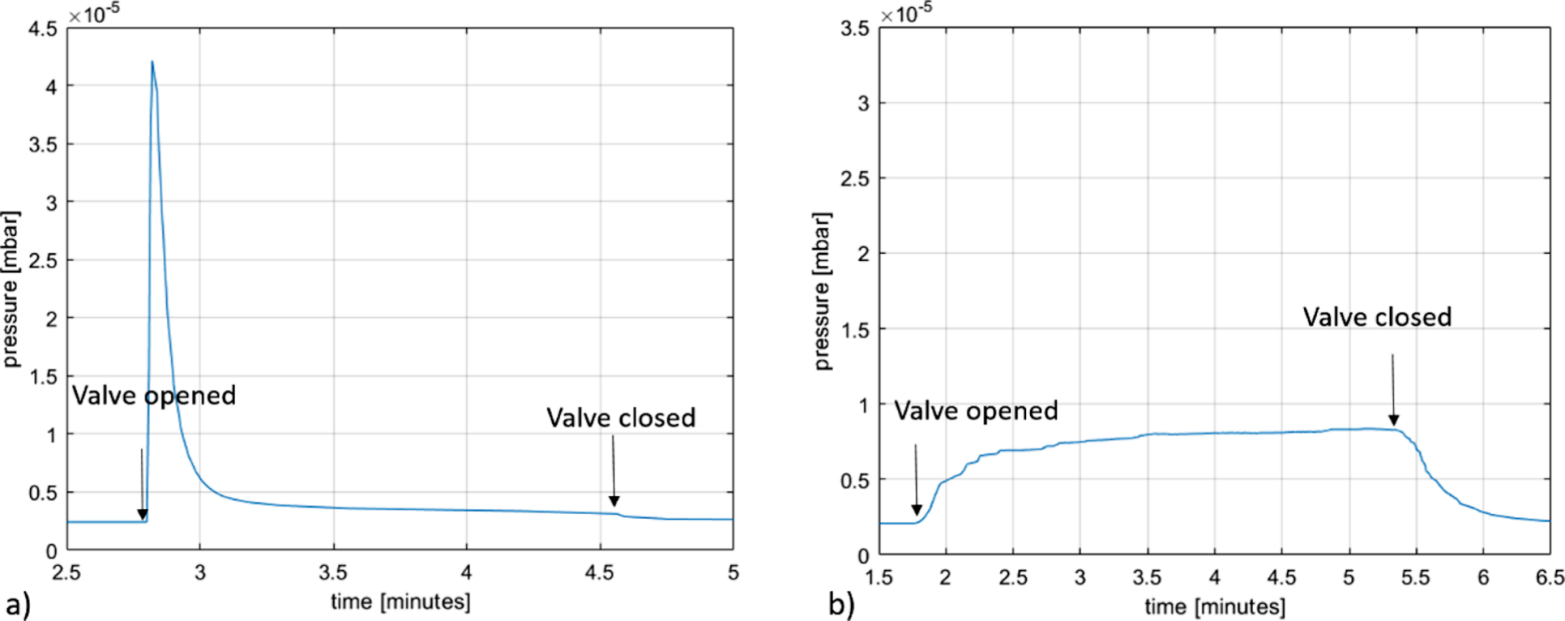
Chamber pressure after opening and closing the GIS valve containing a) Pt(CO)_2_Cl_2_ and b) MeCpPtMe_3_.

Both Pt(CO)_2_X_2_ precursors produced a steep pressure rise upon opening the GIS valve, followed by a somewhat slower pressure drop. Starting at a background pressure of about 2 × 10^−6^ mbar, the pressure rises to about 8 × 10^−6^ mbar upon opening the valve of the MeCpPtMe_3_ GIS and decreases to the background level when the valve is closed. Starting at about the same background pressure, opening the valve of the Pt(CO)_2_Cl_2_ GIS increases the chamber pressure to about 4 × 10^−6^ mbar after an initial sharp pressure rise and subsequent pressure drop. Figure 3a shows the chamber pressure after the valve of the Pt(CO)_2_Cl_2_ GIS was opened and then closed after some time. Almost identical behaviour is observed for Pt(CO)_2_Br_2_. The observation of rapid pressure rise with the Pt(CO)_2_Cl_2_ crucible at 80 °C is consistent with the TGA results in which mass loss corresponding to dissociation of the CO ligands was observed to begin at 80 °C (Figure 1). The sharp pressure increase upon opening the valve may be caused by a partial precursor decomposition in the GIS crucible, resulting in a mixture of precursor molecules and CO [9,11]. When opening the valve, the CO then escapes rapidly, giving rise to a sharp pressure increase in the SEM chamber. When all the CO has left the GIS and the needle, the vapour pressure of the parent molecule remains. This is in sharp contrast with the behaviour of MeCpPtMe_3_, shown in Figure 3b, which does not cause an initial sharp pressure rise in the chamber. This is consistent with TGA data for MeCpPtMe_3_, which do not indicate facile thermal ligand loss reaction such as the loss of CO from metal carbonyls [26].

### Deposition

**Figure 4:**
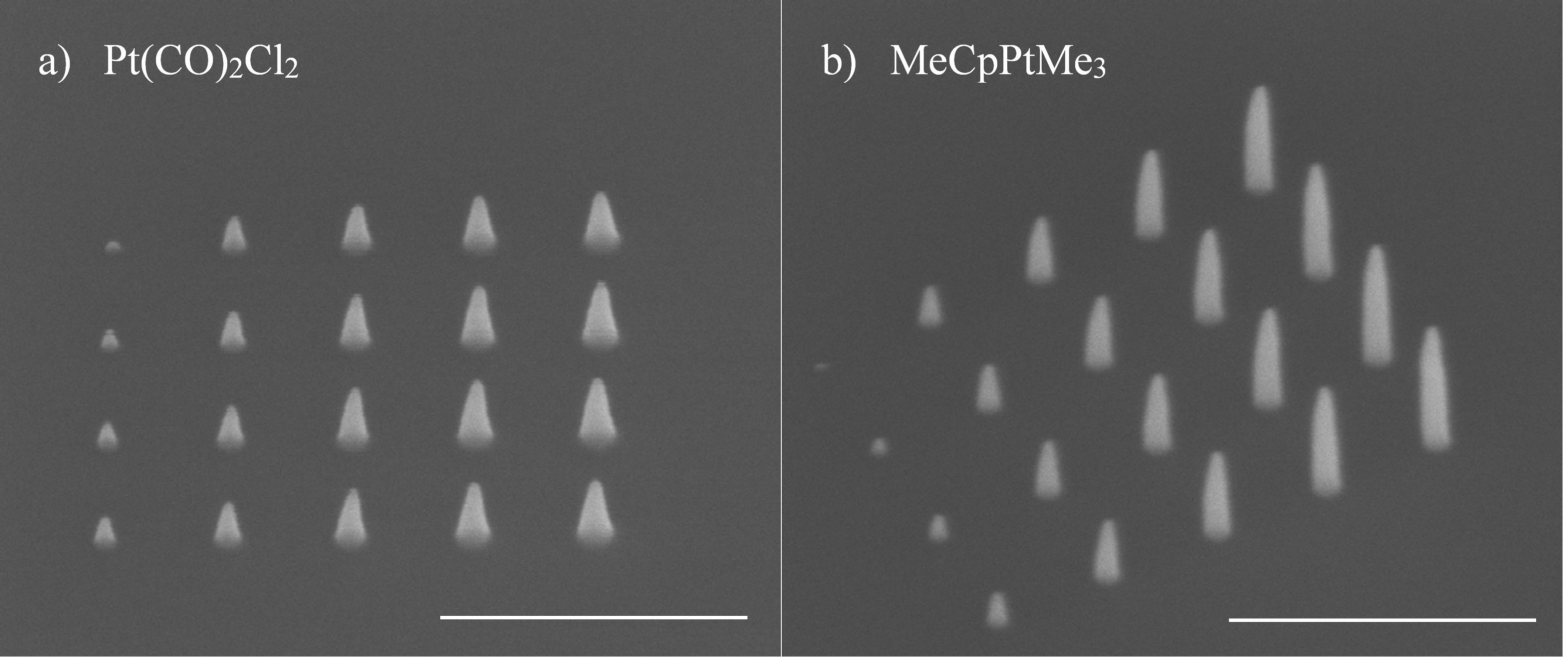
Pillars deposited at 38 pA. a) 35° tilt image of pillars deposited from top to bottom and from left to right from Pt(CO)_2_Cl_2_. b) 35° tilt image of pillars from MeCpPtMe_3_, rotated for better visibility. Scale bars are 500 nm.

**Figure 5:**
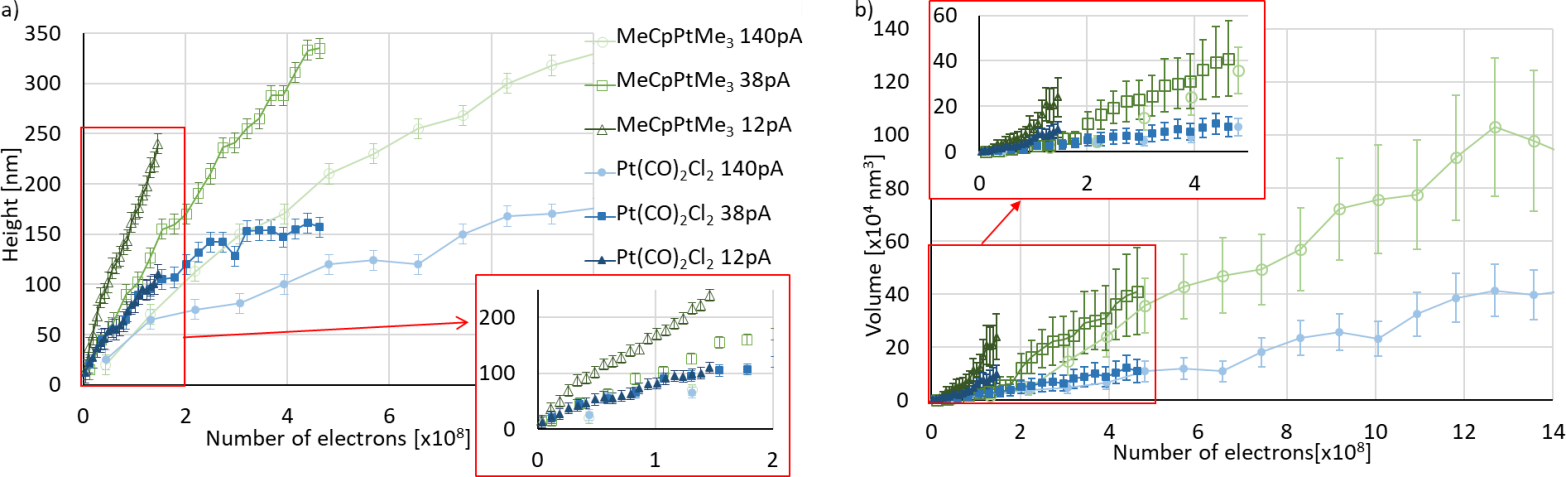
Pillar height a) and volume b) as functions of the electron dose (total number of incident electrons used to grow a pillar), deposited at 12, 38 and 140 pA from MeCpPtMe_3_ (open symbols) and Pt(CO)_2_Cl_2_ (closed symbols). The error bars originate from a ±10 nm error in size measurements taken manually from SEM images. The lines between the points serve as guides to the eye and the insets show expansions of the data in the rectangular (red) areas.

The first set of experiments was aimed at finding the right parameters for deposition in an SEM. Because successful deposition [21], and surface science studies [15,22], from Pt(CO)_2_Cl_2_ were reported already, this precursor was chosen to start with. Because the bromine compound is expected to perform similarly, as mentioned in the Introduction section, it was anticipated that the same set of parameters could be used for the deposition from that precursor. In the first experiment (hereafter referred to as experiment 1), pillars of different height were deposited using point exposures with varying dwell times. The parameters chosen were based on previous studies using MeCpPtMe_3_ that demonstrated growth of visible pillars such that the height could be determined easily [27–28]. A primary beam energy of 18 kV was used with beam currents of 12, 38, and 140 pA. A writing strategy was employed wherein a 4 × 5 array of locations at a 200 nm pitch was exposed in a serial fashion. At each location, the electron beam remained for a different dwell time, starting with 0.5 ms and increasing by 1 ms at each further location. Exposure of each location within the 4 × 5 array is considered a single pass. After each pass, a waiting time of 10 ms was introduced, during which the beam was blanked and the precursor allowed to replenish the area of deposition. This entire process was repeated for 100 passes. Without the inclusion of a waiting time, minimal growth was observed for the first few pillars with the lowest dwell times. Figure 4a shows an array of the resulting pillars. For reference, a similar array of pillars was deposited from MeCpPtMe_3_ using the same parameters (Figure 4b), except for a higher chamber pressure (8 × 10^−6^ mbar). The pillars deposited from Pt(CO)_2_Cl_2_ have a conical shape, and the height is smaller and seems to saturate much more rapidly with electron dose than for the pillars deposited from MeCpPtMe_3_ (Figure 5). Note that in Figure 5 the dose is plotted as the total number of incident electrons used to grow a pillar, that is, the beam current multiplied by the total dwell time at the location of exposure, excluding the waiting time. For point exposures, this is a better-defined measure than the dose per unit area. Although the diameters of the pillars from Pt(CO)_2_Cl_2_, as judged from Figure 4, appear slightly larger than those of pillars deposited from MeCpPtMe_3_, Figure S4 in Supporting Information File 1 shows that they are equal within experimental error. The aspect ratio of the MeCpPtMe_3_ pillars (ca. 6 for the largest pillars) is larger than that of the Pt(CO)_2_Cl_2_ pillars (ca. 2.5 for the largest pillars). The decreasing growth rate (here defined as the increase in height per incident electron, that is, the slope of the curves in Figure 5a) with increasing dose indicates that the growth is still limited by the precursor supply for both precursors, but more so for Pt(CO)_2_Cl_2_ [4,29]. The larger height of the MeCpPtMe_3_ pillars compared to the Pt(CO)_2_Cl_2_ pillars is presumably caused by the higher partial pressure of MeCpPtMe_3_, but may also be caused by many other factors governing FEBID such as the surface residence time, the dissociation cross section, and surface diffusion [30].

From both precursors, Pt(CO)_2_Cl_2_ and MeCpPtMe_3_, arrays of pillars were also deposited at both a lower and a higher beam current of 12 and 140 pA, respectively. Figure 5a displays the heights of those pillars as well. For MeCpPtMe_3_ the growth rate is highest at 12 pA and lowest at 140 pA. This indicates that at 140 pA the growth is severely limited by the precursor supply, whereas at 12 pA the growth tends towards a linear increase with dose, approaching current-limited growth [29]. For pillars deposited from Pt(CO)_2_Cl_2_, the growth rates at 12 and 38 pA are quite similar in the dose range probed in the experiment. At 140 pA the pillar heights are smaller than at lower currents and the growth is clearly limited by the precursor supply. At all currents no linear height increase with dose is observed, which means that the growth is still limited by the precursor supply.

Supporting Information File 1, Figure S4 shows the base diameters of the pillars grown from Pt(CO)_2_Cl_2_ and MeCpPtMe_3_. The diameters of pillars grown from both precursors are largest at 12 pA, presumably due to a larger contribution of precursor surface diffusion to the growth [29]. At low currents, the pillars grow more slowly in height but faster in width; this makes it interesting to plot the deposited volume as a function of the electron dose and compare the growth rates with those at higher currents. Approximating the shape of the pillars either as a cone (shorter pillars) or as a cylinder with a conical top (taller pillars), the pillar volumes were calculated and plotted in Figure 5b. For example, of the deposits in Figure 4a and in Figure 4b, the first two columns are approximated as cones, while the other deposits more closely resemble cylinders with conical tops. It is noted here that the volume of halos deposited around pillars were assumed to be negligible, as no visible halos were observed in the SEM images. At all currents, the volume (Figure 5b) shows a nonlinear initial increase with dose followed by a more linear increase. This reflects the isotropic growth in the early stages of the deposition, when both the diameter and the height increase. At a later stage, when the diameter tends to saturate because secondary electrons generated in the pillar cannot reach the surface to contribute to further lateral growth, the volume increases more or less linearly with height and thus linearly with dose [29]. At this stage pillars grown from MeCpPtMe_3_ grow at a rate approximately 2.5 times faster than those grown from Pt(CO)_2_Cl_2_, regardless of the beam current. This can also be observed for the heights of pillars shown in Figure 5a.

The second set of experiments aimed to grow pillars whose heights vary more linearly with exposure dose. In order to do so, a refresh time equal to ten times the dwell time was introduced immediately after each exposed location, so that the growth should be less limited by the precursor supply. Further, the waiting time between passes was doubled to 20 ms and the number of passes was doubled to 200. This experiment will be referred to as experiment 2. Four pillars were grown at a 200 nm pitch with dwell times of 0.5, 1, 1.5, and 2 ms, from the precursors MeCpPtMe_3_ and Pt(CO)_2_Cl_2_, and now also from Pt(CO)_2_Br_2_, at two beam currents (12 and 38 pA) and a beam energy of 18 kV.

**Figure 6:**
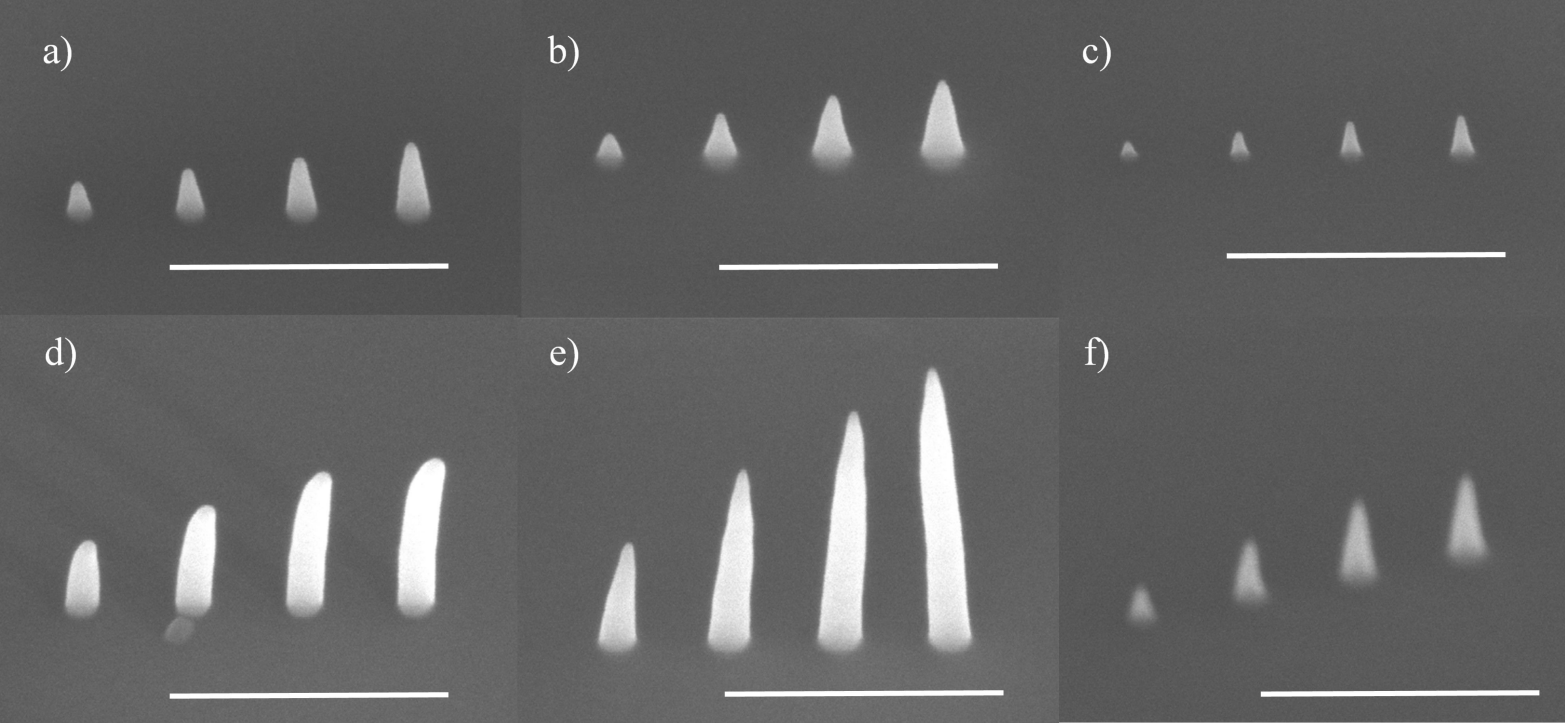
Comparison of pillars grown in experiment 2 from all three precursors a) and d) MeCpPtMe_3_, b) and e) Pt(CO)_2_Cl_2_, and c) and f) Pt(CO)_2_Br_2_. The dwell times for each pillar were 0.5, 1, 1.5 and 2 ms (left to right) and this was repeated for 200 passes. a), b), and c) are deposited at 12 pA. This corresponds to electron doses of 7.5, 15, 22.5, and 30 × 10^6^ electrons. d), e) and f) are deposited at 38 pA, corresponding to electron doses of 24, 47, 71 and 95 × 10^6^ electrons, respectively. All scale bars are 500 nm.

Figure 6 shows SEM 35° tilt images of the pillars deposited at 12 and 38 pA. First, it is observed that deposits can also be grown from Pt(CO)_2_Br_2_, albeit at a lower growth rate than for the other two precursors.

**Figure 7:**
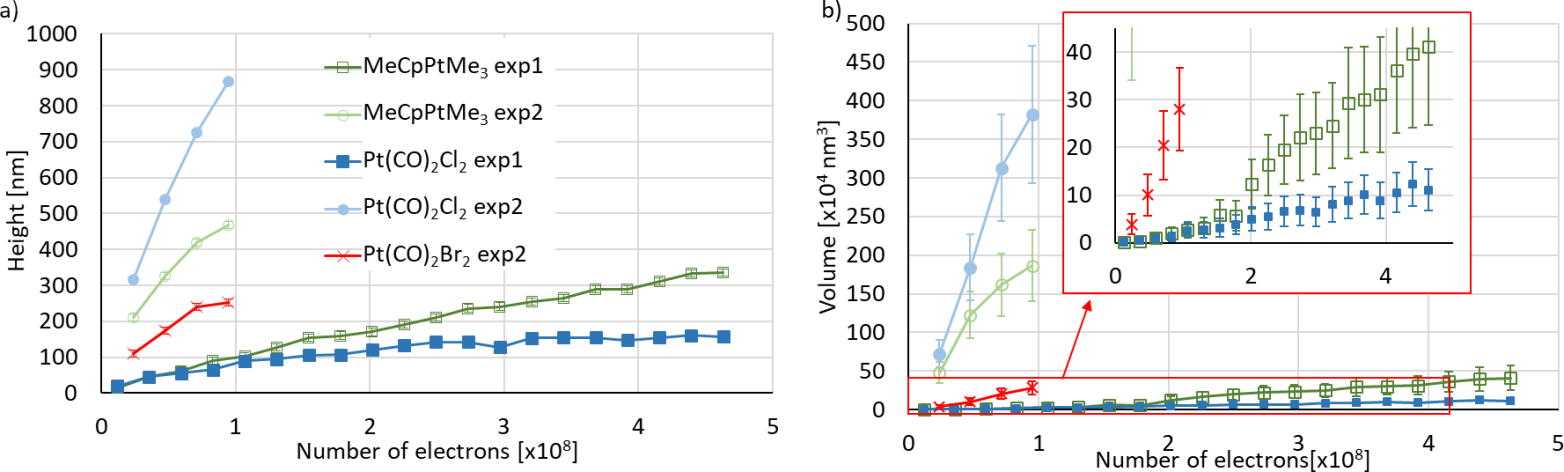
Pillar height a) and volume b) as functions of the number of electrons deposited at 38 pA from MeCpPtMe_3_, Pt(CO)_2_Cl_2_, and Pt(CO)_2_Br_2_. For comparison, the graph also contains the heights obtained from MeCpPtMe_3_ and Pt(CO)_2_Cl_2_ in experiment 1. The error bars originate from ±10 nm errors in size measurements taken manually from SEM images. The lines between the points serve as a guide to the eye and the inset shows an expansion of the data in the rectangular (red) area.

In experiment 1, at 12 pA, the pillars grown from MeCpPtMe_3_ were taller than those grown from Pt(CO)_2_Cl_2_ (see Figure 5a). Conversely, using the deposition strategy of experiment 2, with more time for precursor replenishment, the heights of pillars grown from both precursors at 12 pA were almost the same (Figure 6a and Figure 6b). At 38 pA, however, the pillars grown from Pt(CO)_2_Cl_2_ were taller than those grown from MeCpPtMe_3_ (Figure 6d and Figure 6e). Figure 6b and Figure 6e show the difference in shape and size for pillars deposited from Pt(CO)_2_Cl_2_ at 12 and 38 pA. The heights and volumes of the pillars grown at 38 pA from Pt(CO)_2_Cl_2_ are plotted in Figure 7 and reveal a distinctly enhanced growth with increasing dose via experiment 2 compared to the strategy employed in experiment 1. The results for the other two precursors, as well as the results of experiment 1 using a beam current of 38 pA for comparison, are also included in Figure 7. The pillar diameters are shown in Figure S5 in Supporting Information File 1. We observe that the addition of the refresh time in experiment 2 significantly increased the growth in terms of height, diameter and volume.

Although the purpose of this work was not to find the optimum deposition conditions, the highest growth rates we observed (using experiment 2 at 38 pA) were 0.045 nm^3^/electron (22 e^−^/nm^3^) for Pt(CO)_2_Cl_2_, 0.0035 nm^3^/electron (290 e^−^/nm^3^) for Pt(CO)_2_Br_2_, and 0.020 nm^3^/electron (50 e^−^/nm^3^) for MeCpPtMe_3_. These rates were obtained from the slope of the curves taken at 38 pA in experiment 2 and shown in Figure 7. For all three precursors, the growth was still limited by the precursor flux. To further enhance the growth rate, the local precursor pressure must be increased.

Based on these experiments, we conclude that both new precursors Pt(CO)_2_Cl_2_ and Pt(CO)_2_Br_2_ can be successfully used for FEBID, the former showing faster growth and the latter showing slower growth than the well-known precursor MeCpPtMe_3_.

### Composition

In order to directly compare the composition of the deposits grown from these precursors, square deposits of 250 × 250 nm^2^ were deposited from each precursor in the SEM using the following parameters: 5 kV, 400 pA, 12 ms dwell time, 50 passes, pitch between exposed pixels of 20 nm, and exposure dose of 596 nC/µm^2^. The total deposition time was 100 s.

EDX measurements were performed on these deposits. Typical spectra are shown in Supporting Information File 1, Figure S1. The Pt(CO)_2_Cl_2_ square contained about 55 atom % C and 20 atom % Pt (Supporting Information File 1, Figure S1, top), while the Pt(CO)_2_Br_2_ square had about 65 atom % C and 12 atom % Pt (Supporting Information File 1, Figure S1, middle). These results contrast sharply with previous deposits from Pt(CO)_2_Cl_2_ made in a UHV chamber, which did not contain any carbon and instead consisted of 35 atom % Pt and 65 atom % Cl, as determined from EDX [21]. Deposits from Pt(CO)_2_Br_2_ performed under UHV were similarly composed primarily of platinum and bromine (36 atom % Pt and 55 atom % Br), with minimal carbon and oxygen contamination. For reference, the composition of a square deposited in the SEM from MeCpPtMe_3_ was measured (Supporting Information File 1, Figure S1, bottom) whose composition (18 atom % Pt and 70 atom % C) agreed well with literature values [8–9,31]. It is noted that the deposition conditions in the UHV system and in the SEM were quite different. In the UHV system, deposits from Pt(CO)_2_Cl_2_ were made over 23 h at a chamber pressure between 2 and 7 × 10^−8^ mbar with a beam energy of 3 kV and a beam current of 300 nA, resulting in a deposit size of approximately 20 × 57 µm^2^. Assuming that the deposit size represents the beam size, the current density was 26 mA/cm^2^ and the power density was 78 W/cm^2^. The deposit from Pt(CO)_2_Br_2_ was made in UHV at a pressure of approx. 5 × 10^−8^ mbar, at 3 kV and a beam current of approx. 750 nA, resulting in a deposited area of 4.6 × 10^−2^ mm^2^. The corresponding current density was 1.6 mA/cm^2^ and the power density was 4.9 W/cm^2^. In the SEM, at a chamber pressure of typically 10^−6^ mbar, a square area was exposed with a finely focused beam in a serial fashion (20 nm pitch between pixels) in 50 passes. The current density, calculated as the current per 20 nm diameter pixel, was 127 A/cm^2^ and the corresponding power density was 6.3 × 10^5^ W/cm^2^. Although there is a huge difference in current density between the UHV and the SEM deposition experiments, it is unclear how this would lead to the observed difference in composition. It is more likely that the different vacuum conditions play a crucial role here.

The two potential sources of carbon in the SEM deposits are the precursor molecules themselves or hydrocarbon contamination in the SEM chamber. The latter is clearly absent in the UHV system. To endeavour to reduce the contribution of carbon contamination from the SEM chamber, deposition was done in the SEM after plasma cleaning the chamber. EDX spectra of deposits made after plasma cleaning are shown in Supporting Information File 1 (Figure S2). The composition of all SEM deposits, made before and after plasma cleaning, and the UHV deposits, as determined from EDX, are summarized in Table 1. The Si detected in the spectra is most probably coming from the substrate. The origin of the N is not clear. It could arise from the window of the detector or it could be a Si escape peak caused by the Pt Mα1 photons hitting the detector. The Si Kα escape peak shows up at 1.74 keV below the Pt Mα1 peak [32]. The Pt Mα1 peak is at 2.2 keV and the N peak detected is at about 0.46 keV as shown in Figures S1 and S2 in Supporting Information File 1. Only the composition of the deposits from Pt(CO)_2_Cl_2_ are affected by plasma cleaning, increasing the platinum content by 6 atom % and decreasing the carbon content by the same amount. Although there is a small carbon contribution from contamination, it does not explain the entire carbon content in the deposits. In order to estimate the contamination level of the SEM chamber before plasma cleaning, a deposit was made from contamination only, using the same deposition time as was used for the EDX deposit (100 s), while the heated GIS needle was inserted but with the valve closed. From a tilt image of the deposit, its volume is estimated as 1.4 × 10^7^ nm^3^. The volume of the EDX deposit from Pt(CO)_2_Cl_2_ was approximately 1.67 × 10^8^ nm^3^. Thus, the volume contribution of contamination from the chamber is only about 8.5%. After plasma cleaning the SEM chamber, the contamination test was repeated, but no visible growth of a deposit was observed. This indicates that plasma cleaning successfully removes most of the contamination from the chamber and from the GIS needle as well. To verify that the substrate itself did not contain appreciable amounts of carbon, EDX was performed at a position on the substrate far away from the deposit. The resulting composition was 95.9 atom % Si, only 3 atom % C, and 1.1 atom % O. The corresponding spectrum is shown in Figure S3 in Supporting Information File 1. The fact that no N is seen in this spectrum rules out the possibility that the N originates from the detector window.

**Table 1:** Composition (in atom %), as determined by EDX, of deposits made in the SEM from the three platinum precursors. Deposition was done before (Before) and after (After) plasma cleaning the SEM chamber. The composition of deposits made in UHV were added for comparison.

	Pt(CO)_2_Cl_2_			Pt(CO)_2_Br_2_			MeCpPtMe_3_	

Element	SEM		UHV [21]	SEM		UHV	SEM	
		
	Before	After		Before	After		Before	After

C	55.4	50	—	65.4	66.4	6.5	69.5	67.3
Pt	20.2	25.8	37.6	12.1	11.3	35.9	18.4	18.8
Si	5.4	5.3	3.7	3.7	3.9	1.4	5.8	7.3
O	7.2	6.5	—	10.8	9.7	1.2	6.3	6.6
N	4.3	4.8	—	—	1.6	—	—	—
Br	—	—	—	8	7.0	54.9	—	—
Cl	7.5	7.6	58.7	—	—	—	—	—

An alternative reason for the differences observed between the high vacuum (HV) environment of the SEM and UHV may lie in the presence of water in the SEM. In HV, the deposits are almost halogen free but contain a lot of carbon, whereas in UHV the deposits are almost carbon-free but suffer from a large halogen content.

The water present in HV could play a role in two different ways. First, upon electron irradiation, the water is ionized and could react with the halogen species to form HBr or HCl, both of which are volatile, explaining the low halogen content of the deposits. The remaining reactive OH• radical could react with carbon and convert it to volatile CO. From this reaction, the deposit can lose both the halogen and some carbon content. In this way, water has been used for the purification of FEBID of platinum [33–34] and gold [35]. An analogous reaction occurs with ammonia to get rid of the halogen in deposits from the (η^3^-C_3_H_5_)Ru(CO)_3_Cl precursor [36]. However, this reaction scheme would lead to a much lower carbon content in the deposit than what has been observed. The second possibility is the formation of formic acid and formaldehyde. Recent studies show that electron irradiation of CO and H_2_O does not just lead to the formation of CO_2_, which is volatile, but can also lead to the formation of formic acid and formaldehyde [37]. Both compounds may stay in the deposit and contribute to the observed high carbon content. Detailed reaction schemes can be found in [37]. But this would also lead to a significant amount of oxygen in the deposit, at least as much as carbon if not more. In order to lose this oxygen, the formic acid and formaldehyde should further decompose. At present it is unclear what the reaction mechanisms are that lead to the observed composition of the deposits.

### Post deposition e-beam irradiation

As mentioned above, the deposits grown in the UHV system contained a high percentage of Cl or Br for Pt(CO)_2_Cl_2_ and Pt(CO)_2_Br_2_, respectively [21]. A major motivation for the synthesis of these precursors was the possibility that the halogen ligand could be removed by post deposition e-beam exposure; however, e-beam exposure has been previously shown to remove the halogen only from the surface of deposits [21]. In that study, after exposing a Pt(CO)_2_Cl_2_ deposit to a 3 keV, 300 nA, e-beam in the AES system for 7 h, EDX and AES measurements were performed to evaluate the composition in the bulk and at the surface, respectively. The EDX measurement showed no change in the Pt content while the AES measurement showed an increase from 36 to 56 atom % Pt [21]. This proves that e-beam post deposition exposure removes the Cl from the surface only, and not from the bulk. Indeed, for much thinner deposits created in the AES system post-deposition e-beam irradiation purified deposits to a level of 87% Pt [21]. Thus, it may be useful only for very thin deposits.

The deposits that were grown in the SEM have a much lower halogen content, so the more relevant purification would be to remove the carbon. Nevertheless, we tested the effect of post deposition e-beam exposure on the composition of the SEM-grown Pt(CO)_2_Cl_2_ deposit. The deposit was exposed to a 10 keV, 2.1 nA, e-beam in the SEM, for one hour. This was done before plasma cleaning. Using EDX, no change in the composition was detected, which confirms that the e-beam exposure does not remove Cl from the deposit bulk. Furthermore, no increase was observed in the carbon content, which supports that the carbon in the deposits does not originate from contamination of the SEM chamber.

## Conclusion

The main conclusion of this study is that the two compounds Pt(CO)_2_Cl_2_ and Pt(CO)_2_Br_2_ can both be successfully used as FEBID precursors to make platinum containing deposits.

The shapes of deposits and their growth rates were addressed and compared to deposits made from the commonly used MeCpPtMe_3_ precursor. Although this work did not focus on the optimization of deposition conditions, the highest growth rates we found were 0.045 nm^3^/electron for Pt(CO)_2_Cl_2_, 0.0035 nm^3^/electron for Pt(CO)_2_Br_2_, and 0.020 nm^3^/electron for MeCpPtMe_3_.

Apart from platinum, the deposits grown from the two novel precursors in the SEM, revealed a high carbon content, similar to deposits from the MeCpPtMe_3_ precursor. This is markedly different from deposits grown in UHV, which contained no carbon, but a large halogen content. Contrary to expectations based on volatility, Pt(CO)_2_Br_2_ turned out to perform worse than Pt(CO)_2_Cl_2_ as a FEBID precursor with the lowest growth rate and the lowest platinum content.

Results from this investigation provide added motivation for studies designed specifically to unravel the reasons as to why and how deposition conditions influence the composition of deposits created by FEBID.

## Supporting Information

The file contains EDX spectra of deposits grown from all three precursors before and after plasma cleaning the SEM chamber, an EDX spectrum of the bare Si substrate and graphs of the diameters of pillars grown in experiments 1 and 2.

File 1 Additional experimental data.
