# Supplementary material for: Electron beam-induced deposition of platinum from Pt(CO)2Cl2 and Pt(CO)2Br2
Source: Beilstein J Nanotechnol. 2020 Nov 27;11:1789–800. doi: 10.3762/bjnano.11.161 (PMC7705861; doi:10.3762/bjnano.11.161)
Supplement: File 1 — Additional experimental data. [file Beilstein_J_Nanotechnol-11-1789-s001.pdf]

BEILSTEIN JOURNAL OF NANOTECHNOLOGY

Supporting Information

for

Electron beam-induced deposition of platinum from $Pt(CO)_2Cl_2$ and $Pt(CO)_2Br_2$

Aya Mahgoub, Hang Lu, Rachel M. Thorman, Konstantin Preradovic, Titel Jurca, Lisa McElwee-White, Howard Fairbrother and Cornelis W. Hagen

Beilstein J. Nanotechnol. **2020,** *11,* 1789–1800. doi:10.3762/bjnano.11.161

Additional experimental data

License and Terms: This is a supporting information file under the terms of the Creative Commons Attribution License (https://creativecommons.org/licenses/by/4.0). Please note that the reuse, redistribution and reproduction in particular requires that the author(s) and source are credited and that individual graphics may be subject to special legal provisions.
The license is subject to the *Beilstein Journal of Nanotechnology* terms and conditions: (https://www.beilstein-journals.org/bjnano/terms)

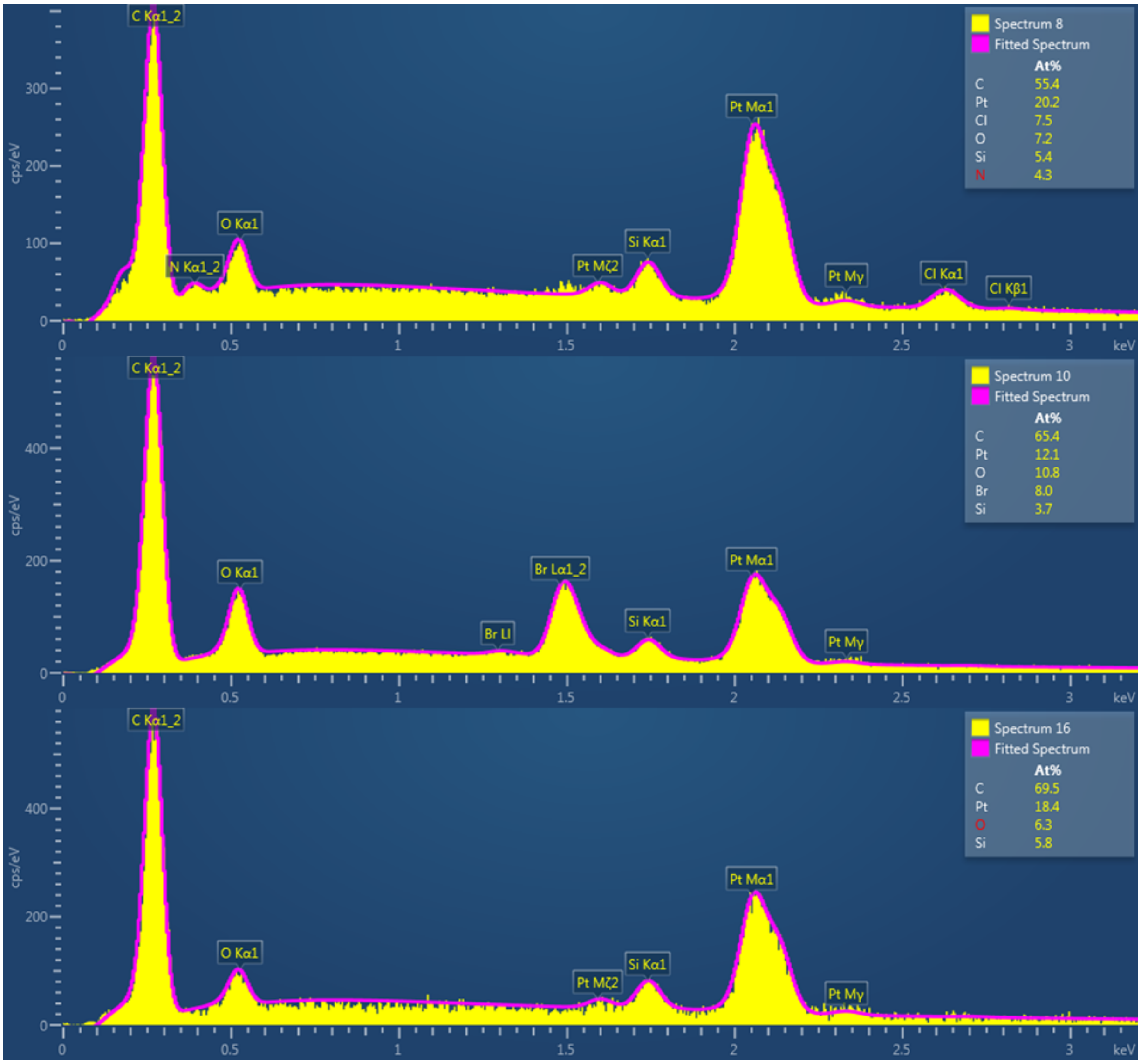

Figure S1: EDX spectra of deposits from the three platinum precursors. Top graph: $PtCl_2(CO)_2$, middle graph: $PtBr_2(CO)_2$, bottom graph: $MeCpPtMe_3$. The deposition was done before plasma cleaning the SEM chamber. All values are in atom %.

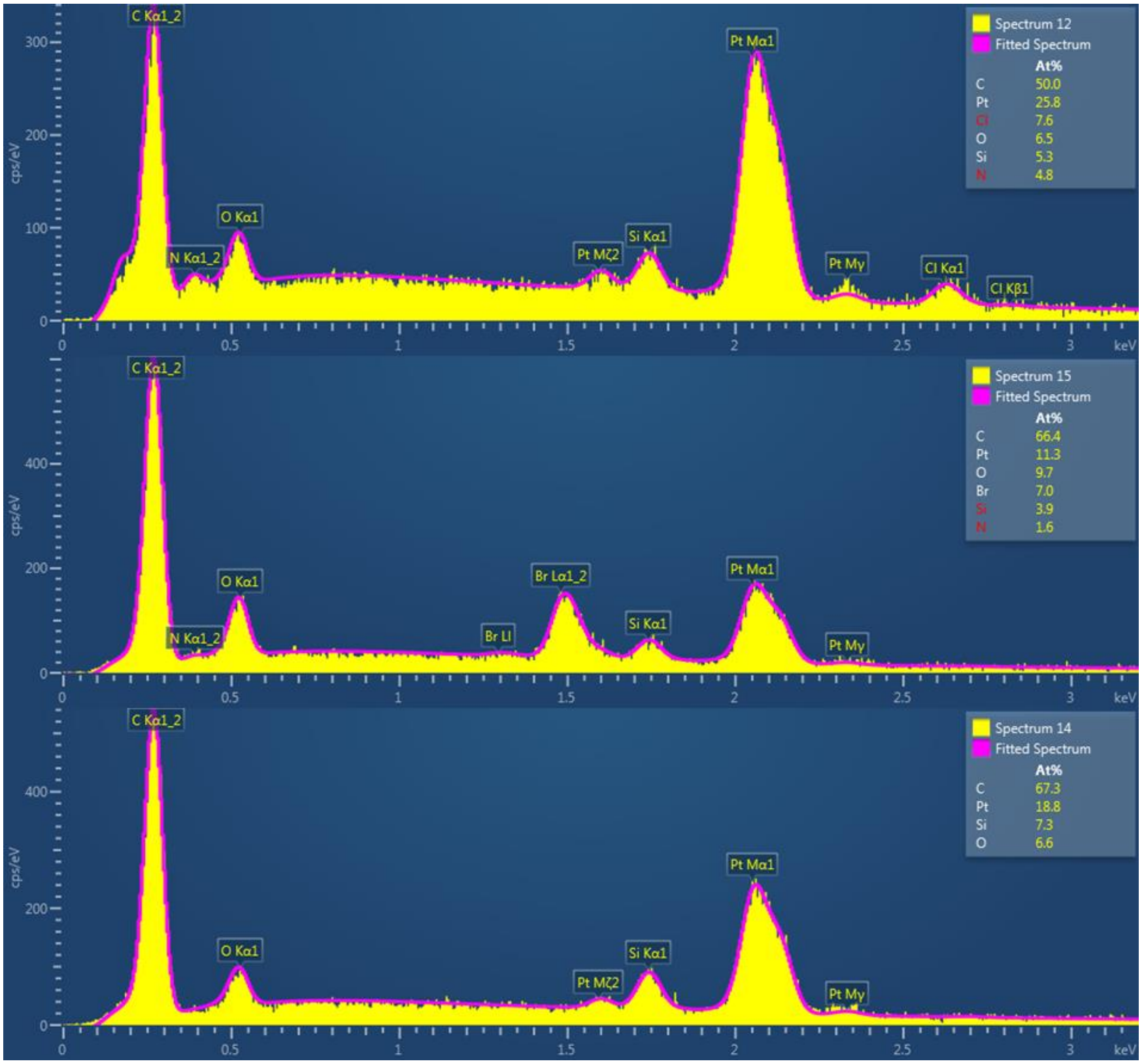

Figure S2: EDX spectra of deposits from the three platinum precursors. Top graph: $PtCl_2(CO)_2$, middle graph: $PtBr_2(CO)_2$, bottom graph: $MeCpPtMe_3$. The deposits were made after plasma cleaning the SEM chamber. All values are in atom %.

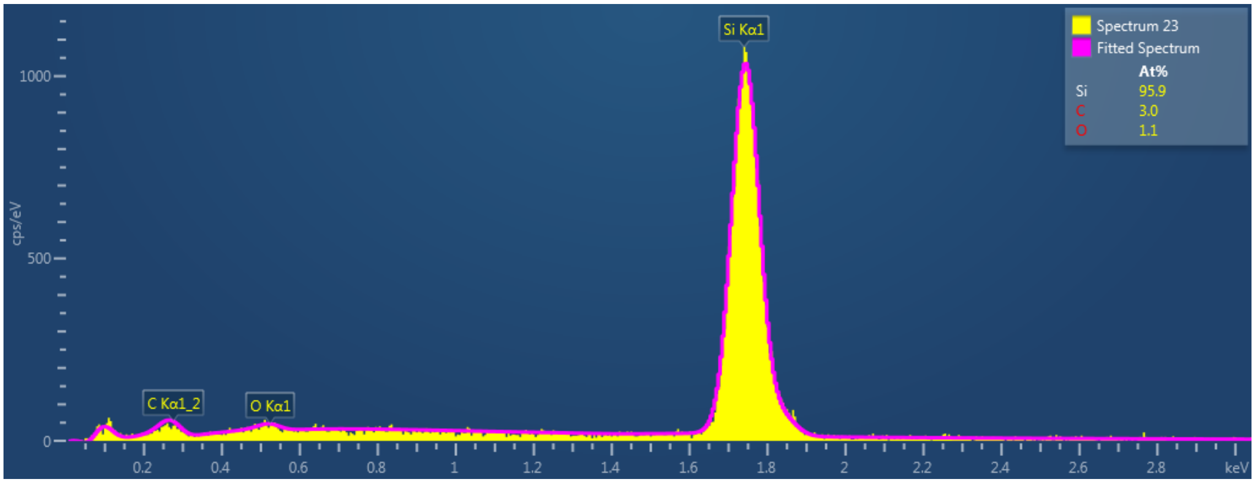

Figure S3: EDX spectrum of the Si substrate. All values are in atom %.

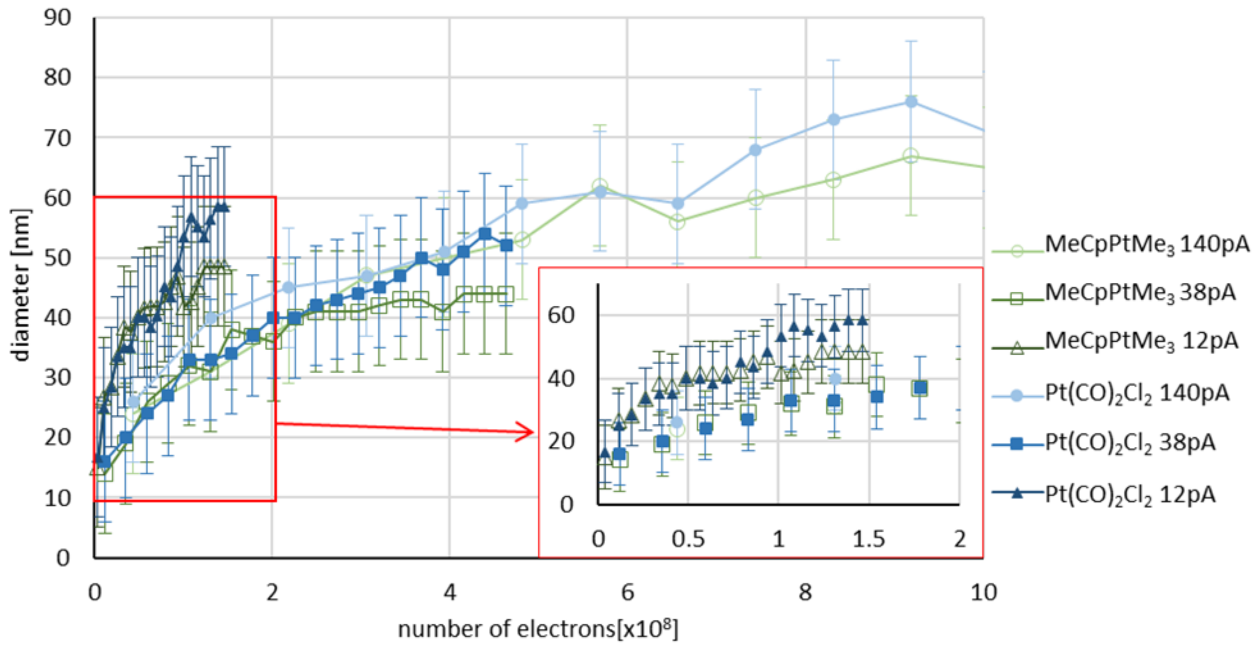

Figure S4: Pillar diameter versus number of electrons per pillar, for pillars deposited at 12, 38 and 140 pA from $MeCpPtMe_3$ (open symbols) and $Pt(CO)_2Cl_2$ (closed symbols). The ±10 nm error bars originate from size measurements taken manually from SEM images. The lines between the points serve as a guide to the eye and the inset shows an expansion of the data in the rectangular (red) area.

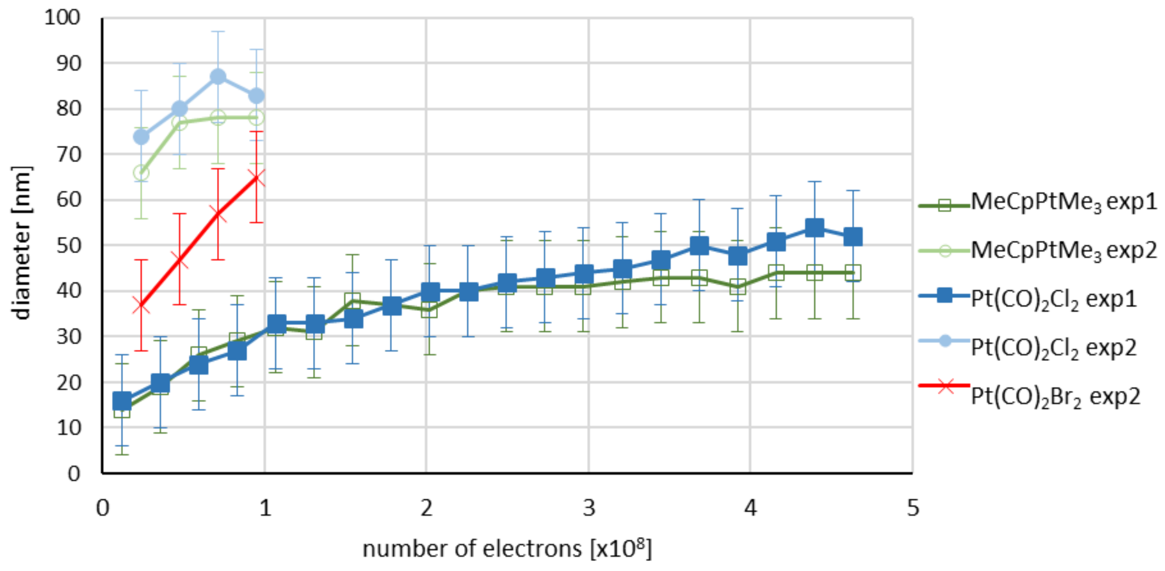

Figure S5: Pillar diameter versus number of electrons per pillar, for pillars deposited at 38 pA from $MeCpPtMe_3$, $Pt(CO)_2Cl_2$ and $Pt(CO)_2Br_2$. For comparison the graph also contains the diameters obtained from $MeCpPtMe_3$ and $Pt(CO)_2Cl_2$ in experiment 1. The ±10 nm error bars originate from size measurements taken manually from SEM images. The lines between the points serve as a guide to the eye.
